# Explore the mechanism of *Astragalus membranaceus* and *Poria cocos* drug pair in improving immunity based on network pharmacology

**DOI:** 10.1097/MD.0000000000038531

**Published:** 2024-06-21

**Authors:** Yuting Bai, Na Ning, Jianjun Zhao, Guoqing Chen, Yuhua Du, Shicong Huang, Xilong Jiang, Xuelan Feng, Yuanyuan Feng, Yi Nan, Ling Yuan

**Affiliations:** a College of Pharmacy, Ningxia Medical University, Yinchuan, China; b Ningxia Chinese Medicine Research Center, Yinchuan, China; c Key Laboratory of Hui Ethnic Medicine Modernization of Ministry of Education, Ningxia Medical University, Yinchuan, China.

**Keywords:** *Astragalus membranaceus* and *Poria cocos* drug pair, immunity, molecular docking, molecular infiltration, network pharmacology

## Abstract

The aim of this study was to investigate the key targets and molecular mechanisms of the drug pair *Astragalus membranaceus* and *Poria cocos* (HFDP) in the treatment of immunity. We utilized network pharmacology, molecular docking, and immune infiltration techniques in conjunction with data from the GEO database. Previous clinical studies have shown that HFDP has a positive impact on immune function. We first identified the active ingredients and targets of HFDP from the Traditional Chinese Medicine Systems Pharmacology database and the Swiss Target Prediction database, respectively. Next, we retrieved the differentially expressed genes (DEGs) related to immunity from the GEO databases. The intersection targets of the drugs and diseases were then analyzed using the STRING database for protein-protein interaction (PPI) network analysis, and the core targets were determined through topological analysis. Finally, the intersection genes were further analyzed using the DAVID database for Gene Ontology (GO) and Kyoto Encyclopedia of Genes and Genomes analyses. Subsequently, by analyzing the expression and prognostic survival of 12 core targets, 5 core target genes were identified, and molecular docking between the hub genes and immunity was performed. Finally, we used the CIBERSORT algorithm to analyze the immune infiltration of immunity genes In this study, 34 effective ingredients of HFDP, 530 target genes, and 568 differential genes were identified. GO and KEGG analysis showed that the intersection genes of HFDP targets and immunity-related genes were mainly related to complement and coagulation cascades, cytokine receptors, and retinol metabolism pathways. The molecular docking results showed that the 5 core genes had obvious affinity for the active ingredients of HFDP, which could be used as potential targets to improve the immunity of HFDP. Our findings suggest that HFDP is characterized by “multiple components, multiple targets, and multiple pathways” in regulating immunity. It may play an essential role in regulating immunity by regulating the expression and polymorphism of the central target genes ESR1, JUN, CYP3A4, CYP2C9, and SERPINE1.

## 1. Introduction

Essentially, immunity is the ability of the human body to identify and eliminate foreign bodies as part of its self-defense mechanism, deal with aging, damage, death, and degeneration of its own cells, and identify and deal with mutant cells and virus-infected cells in the body. Modern medical research has confirmed that immune function is mainly played by the body immune system. The immune system consists of immune organs, immune cells, and immune active substances. These components play a crucial role in identifying and eradicating foreign pathogenic microorganisms, mutated cells, senescent cells, dead cells, and other harmful elements within the body. This process helps maintain overall body health and prevents the onset of different diseases.^[1]^ With the development of the social economy, many unhealthy ways of life and work, such as lack of physical exercise, irregular work and rest, environmental pollution, and an unclean diet, may cause low immunity.^[2]^ Insufficient intake of nutrients (protein, vitamins, trace elements, etc) may also cause a decrease in the body resistance and reduce immune activity, thus having adverse effects on the immune mechanism.^[3]^ The weakening of the body immune system can result in the emergence of different illnesses. Immunological studies have shown that with the increase of age, the body immune function will change to different degrees, and low cellular immune function is one of the important reasons leading to aging and a variety of senile diseases.^[4]^ The function of the immune system is closely linked to the development of various diseases, such as inflammatory disorders, diseases related to weakened immune system, skin conditions, infections, tumors, and more.^[5,6]^ In the field of clinical practice, hormones are frequently employed to suppress the immune response and combat infections in cases of diseases that are infectious or immune-related. However, due to the common side effects of hormone drugs, treatment is limited to a certain extent.^[7]^ However, in Huangdi Neijing (Yellow Emperor Internal Classic), it is stated that “while the zheng qi is inside, the evil can not be dried.” Zheng qi is a functional activity of the human body, including the functions of the viscera and meridians, as well as the ability of the body to resist and repair diseases. When the zheng qi of the human body is strong and the pathogenic toxins are weak, the pathogenic toxin is not easy to invade the human body, or although it is invasive, it does not cause disease. Modern immunology theory and the theory of good and evil in traditional Chinese medicine (TCM) have similar effects.^[8]^ In recent years, the effect of strengthening health and eliminating pathogenic factors in TCM has been very significant in enhancing immunity, showing unique advantages in improving their own immunity and resisting diseases.

Professor Nan Zheng, a TCM master from China fourth batch, has extensive clinical experience in gastric cancer for 50 years. He holds the belief that the development of gastric cancer is caused by a lack of zheng qi in the body and the invasion of pathogenic pathogens, resulting in its occurrence. Taking “supplementing qi and detoxifying” as the therapeutic principle, the *Astragalus membranaceus* and *Poria cocos* decoction (consisting of *A membranaceus, Poria cocos, Ligusticum chuanxiong, cinnamon, Ophiopogon japonicus, Schisandra chinensis, ginger, and jujube*), the ancient prescription of Sun Simiao 22 volume of Essential Prescriptions for Emergency, has been used to treat many patients with gastric cancer undergoing chemotherapy, and remarkable curative effect has been obtained. Both TCM theory research and modern clinical research have shown that numerous Chinese medicines or Chinese medicine extracts possess the ability to enhance the body immunity.^[9]^ *A membranaceus* and *Poria cocos* are both members of the food and medicinal homology. There are few side effects associated with either of these drugs, and they are both suitable for long-term use. Professor Nan Zheng, a specialist in TCM, suggests that, based on extensive clinical experience, this study focuses on investigating the enhancement of immunity through the utilization of 2 components, *A membranaceus* and *Poria cocos*, in the “*Astragalus membranaceus* and *Poria cocos* decoction.”

*A membranaceus* is obtained from the dried root of either *Astragalus membranaceus (Fisch.) Bge.var.mongholicus (Bge.) Hsiao* or *Astragalus membranaceus (Fisch.) Bge.*, both of which are leguminous plants. It is believed that *A membranaceus* enhances the immune system of the human body by increasing yang qi and nourishing the qi of the spleen and lungs. Modern clinical studies have demonstrated that *A membranaceus* has a beneficial impact on the immune function of the body.^[10,11]^ *A membranaceus* contains flavonoids, saponins, polysaccharides, amino acids, trace elements, and other major chemical components,^[12]^ which can promote the specific and nonspecific immunity of the body and have strong immunological pharmacological activity. Immunomodulatory effects of Astragalus polysaccharides on a variety of immune cells. Chun-Xiao Li discovered that Astragalus polysaccharide has the ability to enhance the function of macrophages, natural killer cells, dendritic cells, T lymphocytes, B lymphocytes, and microglia. Additionally, it can stimulate the production of different cytokines and chemokines.^[13]^ The activation of the MyD88-dependent signaling pathway mediated by TLR4 can be regulated by Astragalus polysaccharides, which may impact host immunity.^[14]^ Ma Y found that astragaloside decreased the level of PD-L1 on the cell surface and alleviated PD-L1-related immunosuppression through the miR-135b-5p/CNDP1 pathway.^[15]^ The sclerotium of the Polyporaceae fungus is derived from *Poria cocos (Schw.) Wolf*. The edible medicinal fungus *Poria cocos* has been used in Chinese traditional medicine for over 2 thousand years. Reasons for its usage encompass stimulating urination, enhancing the function of the spleen, and inducing a sense of tranquility. A variety of pharmacological properties are associated with *Poria cocos*, including anti-inflammatory and immune-modulating properties. *Poria cocos* is rich in triterpenoids, polysaccharides, and amino acids, with polysaccharides accounting for over 80% of its content. The main triterpenoids found in *Poria cocos* are wool steroidal tetracyclic triterpenoids.^[16]^ Additionally, *Poria cocos* has been shown to positively regulate the body immune system.^[17–21]^ Tian H discovered that Pachymaran, a component of *Poria cocos*, may exhibit immunomodulatory activity both in vivo and in vitro by activating the TLR4/TRAF6/NF-κB signaling pathway.^[22]^

**Figure 1.**
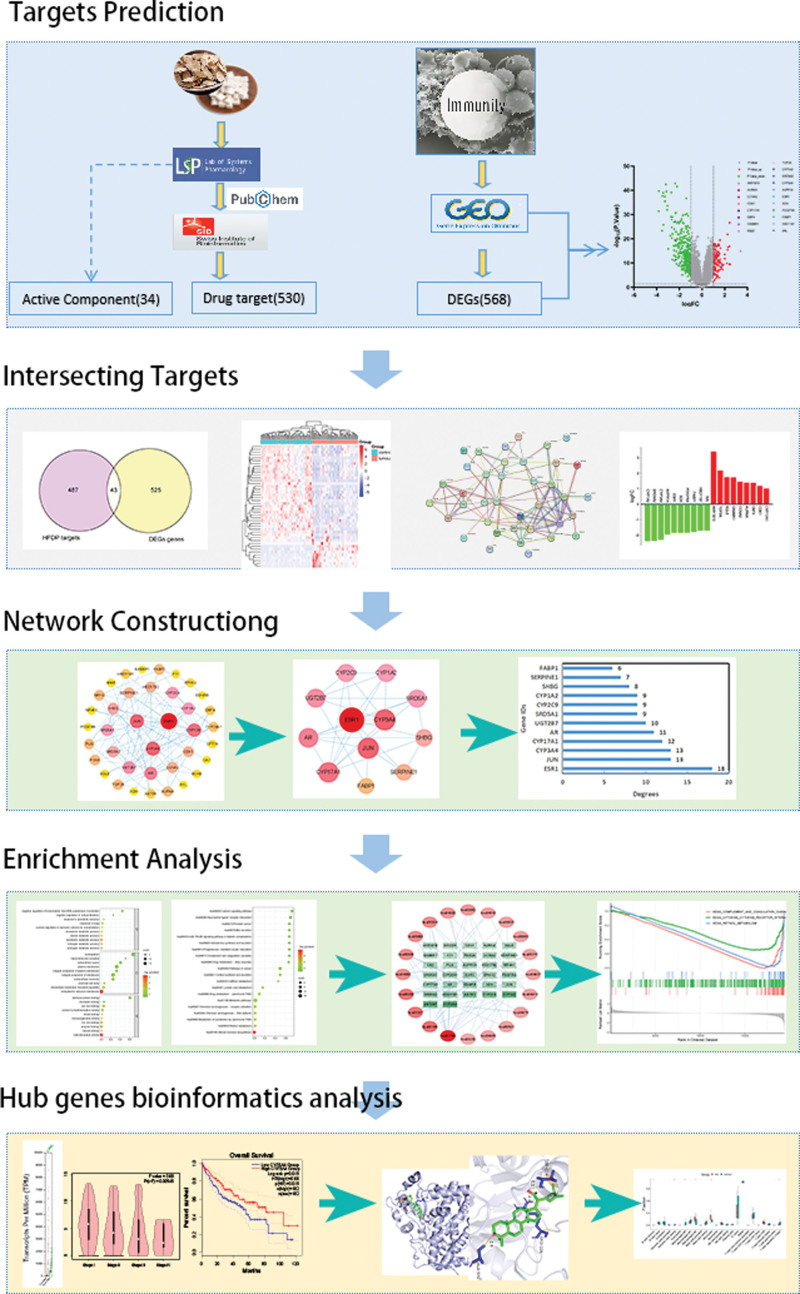
Flowchart of the current research study.

TCM is characterized by a holistic view and dialectical treatment. Disease and syndrome are accompanied by corresponding prescriptions and syndromes, thus the disease-syndrome-prescription combination. A network of disease-gene-target-drug interactions is the basis of network pharmacology. Through systematic observation of the impact and manipulation of medications on the network of diseases, we uncover the enigma of drug synergy in the human organism. The comprehensive and methodical nature of this investigation aligns with the “holistic perspective” theory of TCM and the principle of TCM and its prescriptions’ multi-component, multi-pathway, and multi-target synergistic effect.^[23]^ Utilizing network pharmacology and molecular docking technology, this research acquired the active constituents and targets of *A membranaceu* and *Poria cocos* drug pair (HFDP) from the TCMSP database. Genes that were expressed differently in relation to immunity were acquired by utilizing the term “immunity” in the GEO database. To provide a foundation for clinical application and further fundamental research, an examination of protein interactions, as well as Gene Ontology (GO) and Kyoto Encyclopedia of Genes and Genomes (KEGG) pathway enrichment analyses, was conducted to investigate the primary core targets of HFDP and their connection to enhancing immunity. The flowchart is shown in Figure 1.

## 2. Materials and methods

### 2.1. Screening of related components and prediction of targets in HFDP

Firstly, we used the keywords “*Astragalus membranaceus*” and “*Poria cocos*,” a search was conducted in the Traditional Chinese Medicine Systems Pharmacology Database and Analysis Platform (TCMSP, https://old.tcmsp-e.com/tcmsp.php). Assessing the efficacy of drugs administered orally into the systemic circulation is greatly reliant on oral bioavailability (OB), which stands as a crucial pharmacokinetic characteristic. Drug evaluates the potential of a compound to be a drug. Only molecules with increased OB and drug like (DL) can exhibit excellent pharmacological activity. In “HERB,” specify the screening criteria as having an OB of at least 30% and a DL value of at least 0.18. Retrieve the relevant active ingredients, proceed to download them, and create the active ingredient database. Secondly, enter the active components of the acquired HFDP into the PubChem database (https://pubchem.ncbi.nlm.nih.gov/) in the given order. Finally, find the corresponding Isometric SMILES number for the target prediction, and then input the Isometric SMILES number into the SwissTargetPrediction (http://swisstargetprediction.ch/) perform target prediction, select probability > 0, organize, and remove duplicates to obtain the effective components corresponding to the target points of HFDP.

### 2.2. Screening of immune-related targets

In the GEO database (gene expression omnibus, https://earthobservations.org), we used “immunity” as the keyword to search for immune-related gene chips and “Homo Sapiens” as the research object, the corresponding gene chip GSE174570 was found. To obtain differently expressed genes (DEGs), genes were screened using a standard of *P* < .05 and |LogFC|>1 for up-regulated and down-regulated genes. Utilize the GraphPad Prism application for creating volcano maps and showcasing the findings.

### 2.3. Intersection of disease genes and drug genes

Firstly, by inputting the genes that are expressed differently in the active ingredient targets and immunity of HFDP into the online mapping tool Venny 2.1.0 (https://bioinfogp.cnb.csic.es/tools/venny/index.html), a Wayne diagram can be created to identify the intersection targets. Then, the bioinformatics platform (http://www.bioinformatics.com.cn/) was utilized to generate clustering heatmaps and plot the proportion of DEGs intersecting with drug active ingredient targets. Genes with a screening logFC ≥ 1 were considered up-regulated, while those with logFC ≤ −1 were considered down-regulated. Finally, bar graphs are used to present the results of the intersection, showcasing the top 9 genes that are up-regulated and the top 10 genes that are down-regulated.

### 2.4. The intersection of drug components and target networks in construction

Utilize Cytoscape 3.9.1 software to import the active constituents and common target data of HFDP, creating a network diagram called “drug component - intersection target.” Then, employ Network Analysis and Centiscape 2.2 plug-in tools to conduct network topology analysis. Use parameter Degree, Betweenness, and Closeness to screen for key immune-related active ingredients, and present the Degree of active ingredients in a bar chart.

### 2.5. PPI analysis of protein-protein interactions

The intersection genes between HFDP and DEGs are introduced into the STRING database (https://cn.string-db.org/). The database is configured with the option “Multiple proteins,” the species selected is “Homo sapiens,” and the confidence level is set to medium with a value of “≥ 0.400.” To create a PPI network diagram, the intersecting genes are used to select the concealed non-continuous nodes. To visualize the outcomes, the imported results are utilized in the Cytoscape 3.9.1 application. The PPI network was screened using Cytoscope, and each node was scored using the plug-in CytoNCA. Construct a PPI network graph by filtering the core targets using the Degree, Betweenness, and Closeness parameters. The nodes represent the targets, and the color depth of the nodes represents the contribution value of the targets to the network. Present the core targets in a bar chart based on the degree value.

### 2.6. GO and KEGG enrichment analysis

Perform GO and KEGG enrichment analyses on overlapping targets using the DAVID database (https://david.ncifcrf.gov/). Bubble plots were used to present the top 10 biological processes (BP), cellular components (CC), molecular functions (MF), and the top 20 KEGG signaling pathways, which were selected based on the number of enriched targets. To enhance the understanding of the relationship between signal pathways and targets, a network diagram of pathway targets was created using Cytocape 3.9.1 software, encompassing 20 signal pathways and their enriched targets.

### 2.7. Gene set enrichment analysis (GSEA) enrichment analysis

In order to further investigate the function of immune-related targets, a GSEA was performed on the gene chips obtained from the GEO database using a microbiome platform. The initial genetic material was organized based on logFC measurements, and the gene set chosen for analysis was the “KEGG Gene Sets” specific to humans.

### 2.8. Expression and survival analysis for hub genes

The gene chip study GSE174570 examines the effectiveness and immunoregulation of medications in an experimental model of hepatocellular carcinoma (HCC), leading to the selection of liver cancer as the focus. The 12 genes screened in 2.3 were selected as core genes, and clinical correlation analysis of core targets was performed using GEPAI database (http://gepia.cancer-pku.cn/). Using |LogFC|≥1, *P* < .05 as screening criteria, the expression of core genes in liver cancer and normal liver tissues was analyzed. Generate a “boxplot” in the “expression DIY” column of the boxplots section. Copy the number mapping in the “profile” column. Draw the violin diagram in the “stage plot” of “expression.” Create the predictive analysis diagram in the column labeled “survival plot.”

### 2.9. Molecular docking

In order to assess the dependability of the findings, 5 essential proteins and 8 active ingredients were ultimately selected for molecular docking validation, relying on the screening results of 2.8 central targets and 2.4 crucial active components. Meanwhile, we used the PDB database (https://www.rcsb.org/) to retrieve the 2D configuration of the central protein. Use the PyMOL program to eliminate water and solvent molecules, then save them in PDBQT format after performing hydrogenation, distributing charges, and setting the twist key using the Auto Dock Tool software. Obtain the MOL2 format of active ingredients in TCMSP and save it in PDBQT format after Autodock tools hydrogenation. Autodock tools and Autodock Vina were used to dock the box and calculate the binding energy. The heat map, generated by the online tool on WeChat, displayed the results, while PyMOL software was utilized to visually confirm a portion of the docking outcomes.

### 2.10. Analysis of the immune infiltration correlation

The CIBERSORT (https://cibersortx.stanford.edu/) algorithm in R was used to quantify the fraction of the 22 types of immune cells in the merged data set. We conducted immunoinfiltration analysis of immune cells between the tumor group and the control group. The immune cell types analyzed included M1 macrophages, M2 macrophages, plasma cells, static memory CD4 + T cells, γδ T cells, and mast cells, for a total of 22 kinds. The proportion of immune cells in 22 samples was calculated according to the *P* < .05 de standard. CIBERSORT was used to simulate the transcription feature matrix of immune cells, and the number of calculations was set to 100. After running, the results were imported into the online tool Weishenxin and visualized through the box diagram through the box diagram. It determines the proportion of immune cells in the sample based on the *P* < .05 criterion and utilizes CIBERSORT to simulate the calculation of the immune cell transcription feature matrix. The frequency of calculation is adjusted to 100, and the outcomes acquired upon execution are imported into the online microbiology information tool for visualization using a box plot.

## 3. Result

### 3.1. HFDP components and targets

After conducting a search in the TCMSP database, we acquired a grand total of 34 active components belonging to the HFDP. This includes 20 distinct variations of *A membranaceus* and 15 different types of *Poria cocos*, while MOL000296 was a consensus component (Table 1). Obtain the SMILE numbers of active ingredients using the PubChem database. Using SMILE to search for HFDP-related targets in the Swiss Target Prediction database. Among them, *A membranaceus* has 413 targets, while *Poria cocos* has 254 targets. 530 targets were obtained after removing duplicates.

**Table 1:** Active phytochemicals of *Astragalus membranaceus* and *Poria cocos* drug pair.

Herb name	No.	ID	Phytochemical name	OB(%)	DL
*Astragalus membranaceus*	HQ1	MOL000211	Mairin	55.38	0.78
	HQ2	MOL000239	Jaranol	50.83	0.29
	HQ3	MOL000033	(3S,8S,9S,10R,13R,14S,17R)-10,13-dimethyl-17-[(2R,5S)-5-propan-2-yloctan-2-yl]-2,3,4,7,8,9,11,12,14,15,16,17-dodecahydro-1H-cyclopenta[a]phenanthren-3-ol	36.23	0.78
	HQ4	MOL000354	isorhamnetin	49.6	0.31
	HQ5	MOL000371	3,9-di-O-methylnissolin	53.74	0.48
	HQ6	MOL000378	7-O-methylisomucronulatol	74.69	0.3
	HQ7	MOL000380	(6aR,11aR)-9,10-dimethoxy-6a,11a-dihydro-6H-benzofurano[3,2-c]chromen-3-ol	64.26	0.42
	HQ8	MOL000387	Bifendate	31.1	0.67
	HQ9	MOL000392	formononetin	69.67	0.21
	HQ10	MOL000417	Calycosin	47.75	0.24
	HQ11	MOL000422	kaempferol	41.88	0.24
	HQ12	MOL000433	FA	68.96	0.71
	HQ13	MOL000438	(3R)-3-(2-hydroxy-3,4-dimethoxyphenyl)chroman-7-ol	67.67	0.26
	HQ14	MOL000439	isomucronulatol-7,2’-di-O-glucosiole	49.28	0.62
	HQ15	MOL000442	1,7-Dihydroxy-3,9-dimethoxy pterocarpene	39.05	0.48
	HQ16	MOL000098	quercetin	46.43	0.28
Poria cocos	FL1	MOL000273	(2R)-2-[(3S,5R,10S,13R,14R,16R,17R)-3,16-dihydroxy-4,4,10,13,14-pentamethyl-2,3,5,6,12,15,16,17-octahydro-1H-cyclopenta[a]phenanthren-17-yl]-6-methylhept-5-enoic acid	30.93	0.81
	FL2	MOL000275	trametenolic acid	38.71	0.8
	FL3	MOL000276	7,9(11)-dehydropachymic acid	35.11	0.81
	FL4	MOL000279	Cerevisterol	37.96	0.77
	FL5	MOL000280	(2R)-2-[(3S,5R,10S,13R,14R,16R,17R)-3,16-dihydroxy-4,4,10,13,14-pentamethyl-2,3,5,6,12,15,16,17-octahydro-1H-cyclopenta[a]phenanthren-17-yl]-5-isopropyl-hex-5-enoic acid	31.07	0.82
	FL6	MOL000282	ergosta-7,22E-dien-3beta-ol	43.51	0.72
	FL7	MOL000285	(2R)-2-[(5R,10S,13R,14R,16R,17R)-16-hydroxy-3-keto-4,4,10,13,14-pentamethyl-1,2,5,6,12,15,16,17-octahydrocyclopenta[a]phenanthren-17-yl]-5-isopropyl-hex-5-enoic acid	38.26	0.82
	FL8	MOL000287	3beta-Hydroxy-24-methylene-8-lanostene-21-oic acid	38.7	0.81
	FL9	MOL000289	pachymic acid	33.63	0.81
	FL10	MOL000290	Poricoic acid A	30.61	0.76
	FL11	MOL000291	Poricoic acid B	30.52	0.75
	FL12	MOL000292	poricoic acid C	38.15	0.75

DL = drug like, OB = oral availability.

### 3.2. GEO gene chip and differential gene analysis

Choose the gene chip set labeled as GSE174570 in the GEO database, and perform the analysis of the selected gene expression profile data in the GEO database using the GEO2R function analyze. We have a total of 19,444 genes. The gene chip has a total of 114 samples, including 57 healthy samples as the control group and 57 liver cancer groups as the tumor group. By using the criteria of *P* < .05 and | logFC |>1, we screened and obtained a significant total of 568 genes that showed differential expression. Among these genes, there were 140 up-regulated genes and 428 down-regulated genes.

### 3.3. Intersection of DEGs and drug targets

**Figure 2.**
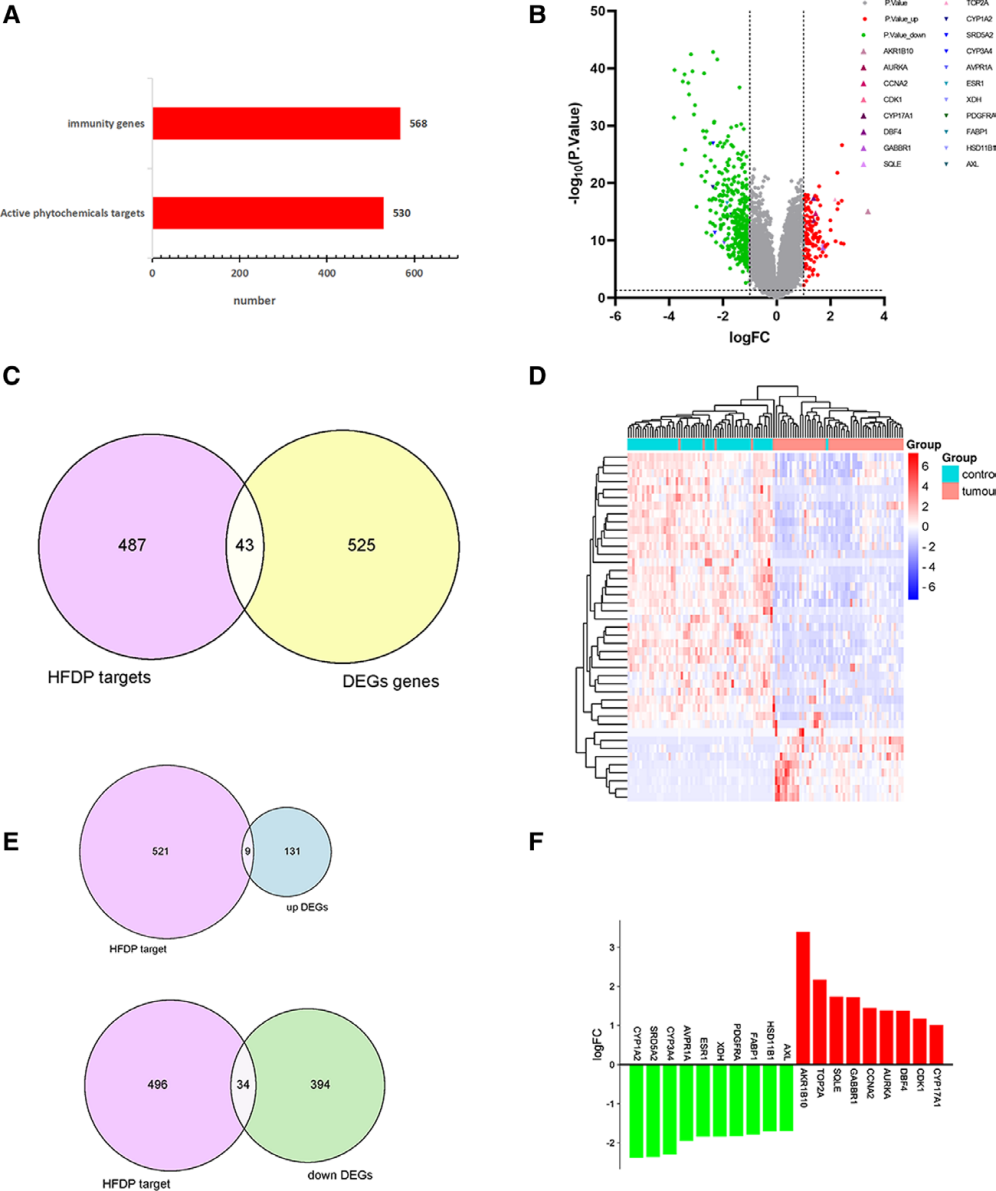
DEGs and drug target acquisition. (A) Bar graph of potential protein targets of active phytochemicals in HFDP and regulated genes of DEGs. (B) The volcano of DEGs. (C) Venn diagram of the intersection of the HFDP target and immunity genes. (D)The heat map of intersection genes. (E) Intersection of up-regulated and down-regulated genes of DEGs by HFDP drug targets respectively. (F) Bar graph of 19 down-regulated and up-regulated genes. DEGs = differentially expressed genes, HFDP = *Astragalus membranaceus* and Poria cocos drug pair.

In Figure 2A, display a bar graph illustrating the quantity of bars for 568 genes with differential expression and 530 targets in HFDP. Intersect the drug pair targets with differently expressed genes and plot them using the online mapping tool Venny 2.1.0, resulting in 43 intersecting targets, as shown in Figure 2C. Import 43 intersecting targets into the online mapping tool of bioinformatics and draw a clustering heatmap, as shown in Figure 2D. Compare the genes that are up-regulated and down-regulated with the target of HFDP, and create a Venny plot using Venny2.1.0. Figure 2E displays the obtained results, indicating that there were 9 intersection genes up-regulated and 34 intersection genes down-regulated. Import DEGs into GraphPad Prism, draw a volcanic map, and label the top 9 up-regulated intersection genes and the top 10 down-regulated intersection genes, as shown in Figure 2B. Incorporate the highest 9 intersection genes that are up-regulated and the highest 10 intersection genes that are down-regulated into the field of bioinformatics. Subsequently, generate a histogram illustrating the up-regulated and down-regulated genes, as depicted in Figure 2F.

### 3.4. PPI network construction and recognition of hub genes

**Figure 3.**
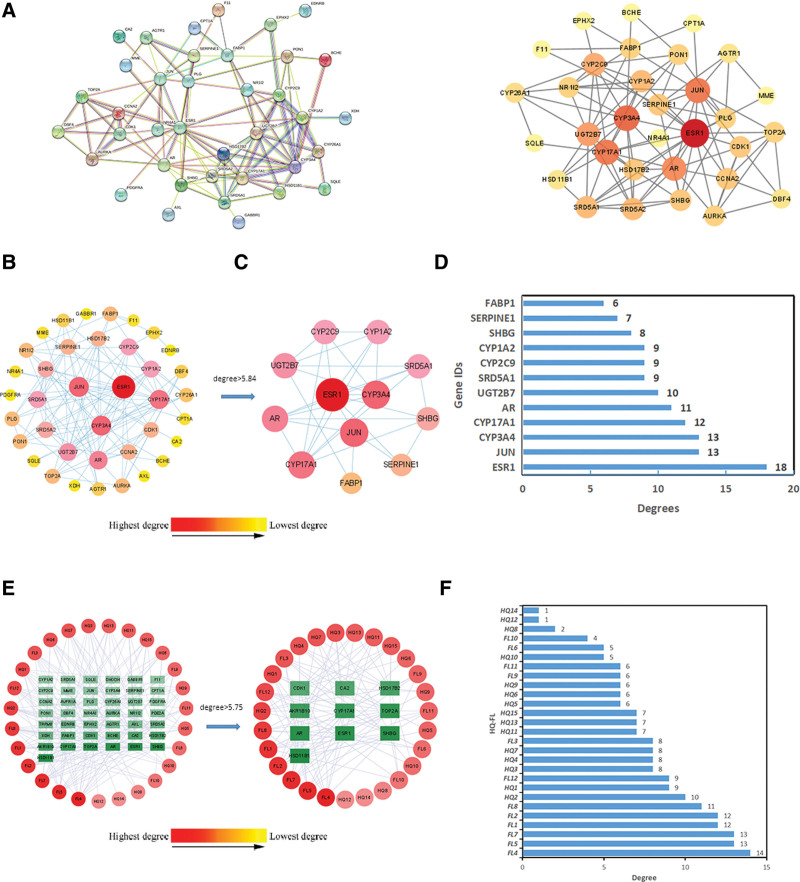
Screening core components and core objectives of HFDP in the treatment of immunity. (A) Intersection PPI network interaction map constructed based on STRING website. (B) The PPI network of the 43 intersecting targets was constructed using Cytoscope software. (C) Screening the top 12 core genes in the network according to the degree. (D) The degree of top 12 core genes was drawn by bar graph. (E) Interaction network diagram of component-intersection targets constructed based on Cytoscape 3.9.1 software. (F) The histogram shows the degree value of the top 27 core components. HFDP = *Astragalus membranaceus* and *Poria cocos* drug pair, PPI = protein-protein interaction.

Firstly, we upload 43 intersection targets to the STRING database for PPI analysis. According to the findings, the network consists of 43 nodes and 111 edges, with an average of 5.16 nodes and a local clustering coefficient of 0.497 on average. Set hidden discontinuous nodes, as shown in Figure 3A. We used the plug-in CytoNCA to perform a topology analysis of the PPI network, as shown in Figure 3B. Core targets were screened using the parameters Degree, Betweenness, and Closeness, and 12 immune-related core targets were selected with a Degree value > 5.84, as shown in Figure 3C. The node color changes from red to yellow, indicating a gradual decrease in Degree value. Draw a bar chart for the degree values of 12 core targets, as shown in Figure 3D. We got the core targets such as ESR1, JUN, CYP3A4, CYP17A1, AR, UGT2B7, SRD5A1, CYP2C9, CYP1A2, SHBG, SERPINE1, and FABP1, and the network diagram was obtained in Figure 3D.

### 3.5. Construct an interaction network between HFDP active ingredients and targets

To create the interaction network diagram, Figure 3E displays the incorporation of 34 active components and 43 shared targets from the medicinal combination of *A membranaceus* and *Poria cocos* in Cytoscape 3.9.1 software. The network shows 70 nodes and 204 edges. The Cytoscape plug-in CytoNCA was utilized for topology analysis to screen core components based on the parameters Degree, Betweenness, and Closeness. Ten core targets were screened with a Degree value > 5.75. The nodes change from red to yellow, indicating a gradual decrease in the Degree value. Figure 3F displays a bar chart illustrating 34 active ingredients. Eight potential HFDP core components: FL4 (Cerevisterol), FL5 ((2R)-2-[(3S,5R,10S,13R,14R,16R,17R)-3,16-dihydroxy-4,4,10,13,14-pentamethyl-2,3,5,6,12,15,16,17-octahydro-1H-cyclopenta[a]phenanthren-17-yl]-5-isopropyl-hex-5-enoic acid), FL7 ((2R)-2-[(5R,10S,13R,14R,16R,17R)-16-hydroxy-3-keto-4,4,10,13,14-pentamethyl-1,2,5,6,12,15,16,17-octahydrocyclopenta[a]phenanthren-17-yl]-5-isopropyl-hex-5-enoic acid), FL1 ((2R)-2-[(3S,5R,10S,13R,14R,16R,17R)-3,16-dihydroxy-4,4,10,13,14-pentamethyl-2,3,5,6,12,15,16,17-octahydro-1H-cyclopenta[a]phenanthren-17-yl]-6-methylhept-5-enoic acid), FL2 (trametenolic acid), FL8 (3beta-Hydroxy-24-methylene-8-lanostene-21-oic acid), HQ2 (Jaranol), and HQ1 (Mairin) were selected for molecular docking studies.

### 3.6. GO and KEGG enrichment analysis

**Figure 4.**
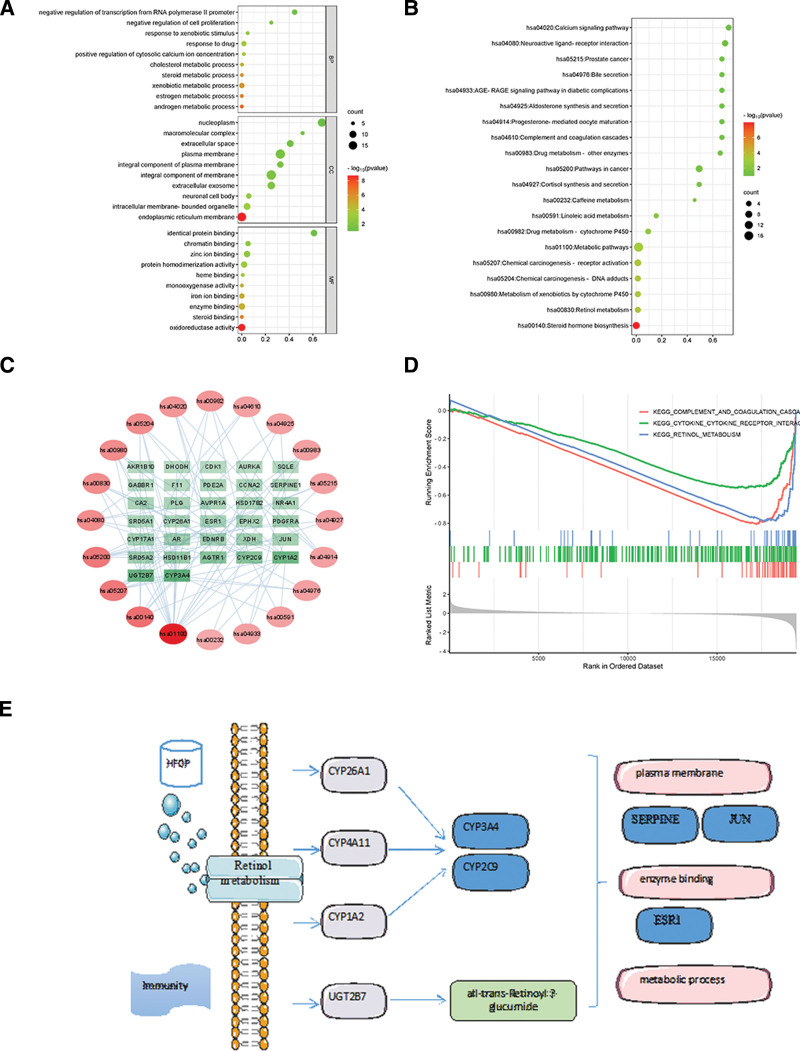
GO and KEGG enrichment analysis. (A) GO enriched analysis of HFDP treatment of immunity, including BP, MF, and CC. (B)KEGG enriched analysis of HFDP treatment of immunity. (C)PPI network diagram of KEGG pathway and core gene. (D)GSEA plot based on GSE174570. (E)Molecular mechanism diagram of core target therapy for immunity. BP = biological process, CC = cellular component, MF = molecular function, GO = gene ontology, GSEA = gene set enrichment analysis, HFDP = *Astragalus membranaceus* and Poria cocos drug pair, KEGG = Kyoto Encyclopedia of Genes and Genomes, PPI = protein-protein interaction.

The DAVID database imported 43 targets that intersected for GO enrichment analysis, leading to a total of 95 BP, 17 cell components (CC), and 33 MF. Arrange GO-BP, GO-CC, and GO-MF in ascending order according to the number of enriched targets (Count value), select the top 10 pathways, and use the microbiome online mapping tool to draw bubble plots, as shown in Figure 4A. The findings indicate that the intersecting genes primarily participate in various BP, including metabolism of foreign substances, response to medication, inhibition of transcription from the RNA polymerase II promoter, metabolism of androgens, metabolism of estrogens, metabolism of steroids, metabolism of cholesterol, regulation of cytosolic calcium ion concentration, response to foreign substance stimuli, inhibition of cell proliferation, etc. Additionally, the CC mainly consist of membrane integral components, plasma membranes, endoplasmic reticulum membranes, nucleoplasm, extracellular exosomes, intracellular membrane-bound organelles, extracellular spaces, integral components of plasma membranes, neuronal cell bodies, macromolecular complexes, etc. Furthermore, the MF primarily involve activities such as oxidoreductase, enzyme binding, protein homodimerization, zinc ion binding, identical protein binding, iron ion binding, chromatin binding, steroid binding, monooxygenase activity, heme binding, etc.

By performing KEGG enrichment analysis on a set of 43 overlapping genes, we identified a grand total of 20 pathways that are associated with these genes. The enriched targets (count value) were used to arrange them in ascending order, and the top 20 pathways were chosen to create bubble plots using the microbiome online mapping tool, as depicted in Figure 4B. The KEGG pathway primarily consists of metabolic pathways, biosynthesis of steroid hormones, activation of receptors in chemical carcinogenesis, cancer pathways, metabolism of retinol, DNA adducts in chemical carcinogenesis, metabolism of xenobiotics by cytochrome P450, interaction of neuroactive ligands with receptors, cytochrome P450-mediated drug metabolism, and the calcium signaling pathway. This suggests that the immune system may be strengthened by the active components of the HFDP through the aforementioned mechanisms.

To create the target pathway interaction network diagram in Cytoscape 3.9.1 software, import the top 20 KEGG pathways and 43 intersection targets, as depicted in Figure 4C. We obtained 12 key genes ESR1, JUN, CYP3A4, CYP17A1, AR, UGT2B7, SRD5A1, CYP2C9, CYP1A2, SHBG, SERPINE1, and FABP1, indicating that these genes are involved in various pathways that are indispensable in enhancing immunity, as shown in Figure 4E.

### 3.7. GSEA based on GSE174570

Examine the gene collection of gene chip GSE174570 through GSEA analysis on the WeChat online mapping tool, as depicted in Figure 4D. The findings indicated that the gene chip targets are associated with immunity via complement and coagulation cascades, cytokine receptors, and retinol metabolism pathways.

### 3.8. Clinical data analysis

**Figure 5.**
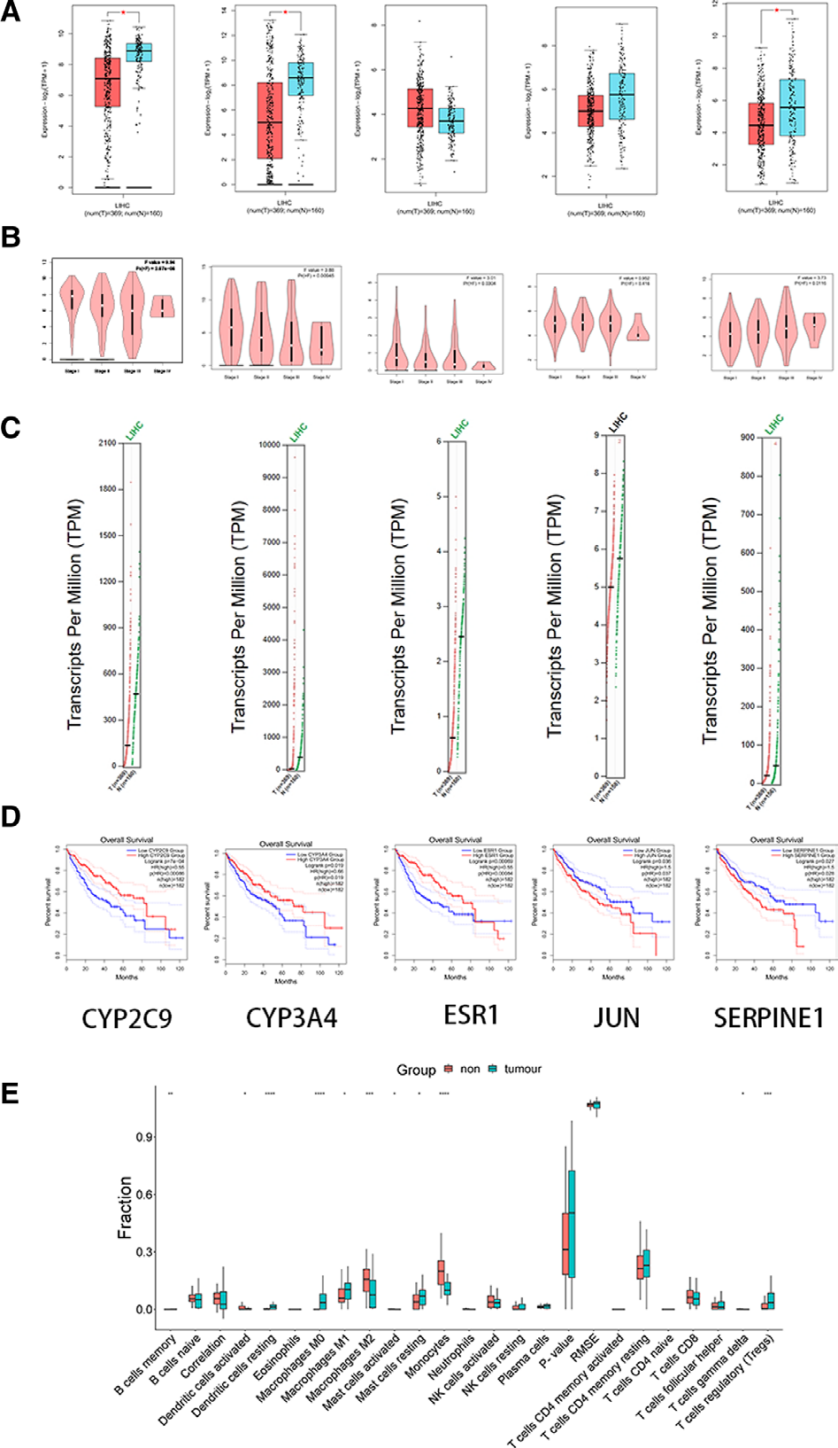
Immune infiltration. (A)Box diagram of clinical relevance analysis of core targets based on GEPAI database. (B)Violin plot of clinical relevance analysis of core targets based on GEPAI database. (C)Copy number diagram of clinical relevance analysis of core targets based on GEPAI database. (D) Prognostic analysis map of clinical correlation analysis of core targets based on GEPAI database. (E) Immunoinfiltration analysis diagram.

The GEPIA database was used to import 12 core targets for clinical correlation analysis, with liver cancer (LIHC) being chosen as the specific cancer type. Based on *P* < .05, 5 genes were ultimately identified, namely ESR1, JUN, SERPINE1, CYP2C9, and CYP3A4. In the group of liver cancer patients, the findings indicated that the expression levels of ESR1, SERPINE1, CYP2C9, and CYP3A4 were significantly reduced. Liver cancer patients with high expression of ESR1, CYP2C9, and CYP3A4 exhibited a greater overall survival curve compared to those with low expression. Conversely, patients with high expression of SERPINE1 and JUN had a lower overall survival curve than those with normal expression. Refer to Figures 5A through D.

### 3.9. Immune infiltration

The CIBERSORT algorithm was utilized to analyze GEO data and calculate the percentage of 22 different immune cell types present in the sample. It was found that the abundance of 11 immune cells had significantly changed (*P* < .05) through boxplot analysis. Significant differences were observed in B cell memory, activated dendritic cells, Macrophages M0, Macrophages M1, Macrophages M2, activated mast cells, resting mast cells, monocytes, gamma delta T cells, and regulatory T cells (Tregs) (Fig. 5E). These findings indicate that these 11 types of immune cells play a very important role in the occurrence and development of immunity.

### 3.10. Molecular docking

**Figure 6.**
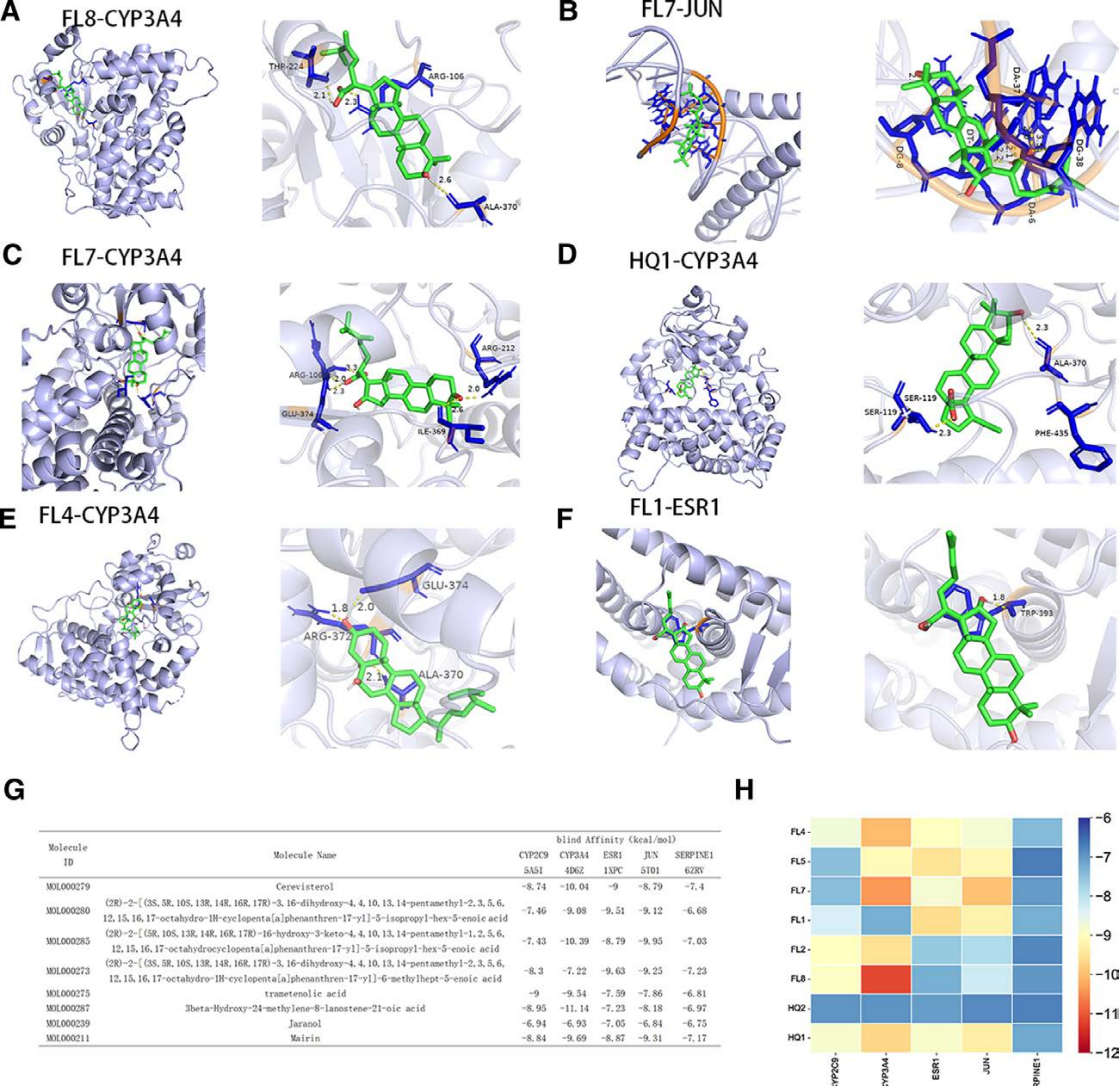
Molecular docking. (A–F) The molecules and drugs with the lowest docking energies are visualized. (G) The docking energy of 5 core targets and 8 core components in 3-line table. (H) Molecular docking energy heat map of 5 core targets and 8 core components.

By analyzing the expression and survival prognosis of core genes in liver cancer, we selected 5 core genes with significant statistical differences to perform molecular docking verification with the first 8 active components of HFDP. Through the use of PDB database, we determine the corresponding to the center of target genes protein crystals. PDB with ESR1, JUN, SERPINE1, CYP2C9, and CYP3A4 crystal structures are 1XPC, 5T01, 6ZRV, 5A5I, and 4D6Z. The binding energy < -7kcal.mol-1 represents a high affinity for receptors and ligands, as well as a high docking activity and a high likelihood of interaction. According to the results, as illustrated in Figure 6G and H, the 8 active components have favorable binding capacities with 5 crucial targets. The interaction strength between FL8 and CYP3A4 is the least at −11.14 kcal.mol-1. We visualized the docking of some active ingredients with the lowest binding energy and proteins, as shown in Figure 6A to F.

## 4. Discussion

In this study, a total of 34 effective ingredients of HFDP were obtained by using the TCMSP database, including 20 kinds of *A membranaceus*, 15 kinds of *Poria cocos*, and 530 targets. A total of 568 differentially expressed immune-related genes with 43 intersection targets were identified by using the keyword “immunity” in the GEO database. The analysis of PPI, GO, and KEGG pathway enrichment of the common targets revealed their predominant involvement in immune enhancement therapy via metabolic pathways, modulation of cell membrane matrix, engagement in enzyme activity, participation in diverse BP, regulation of retinol metabolism, complement and coagulation cascade, as well as interaction among cytokines. The screening of PPI core target genes revealed that the HFDP includes 12 core targets, such as ESR1, JUN, CYP3A4, CYP17A1, AR, UGT287, SRD5A1, CYP2C9, CYP1A2, SHBG, SERPINE1, FABP1, etc, which are beneficial for enhancing immunity. These core genes can be directly or indirectly involved in metabolism, steroid hormone synthesis, etc, so as to improve immunity. Among them, CYP2C9, CYP26A1, CYP1A2, CYP3A4, and UGT2B7 are related to metabolic pathways.

Through core target analysis, we found that ESR1 serves as a crucial catalyst for breast cancer that is positive for estrogen receptors, and alterations in the ligand-binding domain of ESR1 significantly contribute to the development of acquired resistance to endocrine therapy, particularly aromatase inhibitors.^[24–26]^ According to Hu X, the fusion of estrogen receptor 1 (ESR1) with the HS1BP3 promoter was discovered to have the ability to hinder HCC proliferation and enhance prognosis.^[27]^ Sun Z found that the mouse TSG101 gene, or a segment of it, hinders ligand-induced activation of nuclear receptors like AR and ESR, crucial for prostate and breast cancer.^[28]^ Moreover, Yang C noted that AR diminishes the functionality and aridity of male tumor-infiltrating CD8 + T cells through modification of epigenetic and transcriptional pathways. This underscores the significance of gender-specific CD8 + T stem cells in cancer progression and immune response to therapy.^[29]^ In liver tumors, Chang TJ research discovered that the androgen receptor (AR) exhibited the highest expression level within the steroid receptor family. Furthermore, the ARCAP protein was identified as a co-regulator capable of interacting with AR in the liver, with high expression observed in both liver cancer cell lines and adjacent tumors.^[30]^ Samarkina A investigation revealed a notable elevation in AR expression within BRAFi resistant melanoma cells, as well as in sensitive cells following brief exposure to BRAFi.^[31]^ Feng L found that SERPINE1 exhibits high expression in gastric cancer, which is linked to an unfavorable prognosis. SERPINE1 may regulate the copper sag and immune microenvironment through a series of pathways.^[32]^ Mafra RP demonstrated that SERPINE1 could potentially be linked to venous thrombosis in individuals diagnosed with pancreatic cancer.^[33]^ Li discovered that SERPINE1, apart from its crucial involvement in cell adhesion, migration, and invasion, can also stimulate tumor vascularization, thereby facilitating cell scattering and tumor spread.i^[34]^ Y Abiko found that ST induced apoptosis of oral squamous cell cancer cell line SAS cells probably occurs through the c-fos and c-jun pathways.^[35]^ Priyadarshini demonstrated that BLM has the ability to act as a suppressor of tumor growth by promoting the transformation of c-JUN, thus inhibiting its role as a proto-oncogene.^[36]^ Numerous endogenous and exogenous substances can be metabolized by Cytochrome P450 enzymes. Most anticancer drugs are metabolized by the CYP3A subfamily, particularly the CYP3A4 subtype.^[37]^ The gene CYP3A4 is involved in the metabolism of vitamin D, and research has indicated that vitamin D plays a crucial role in the treatment of several types of cancer.^[38]^ Hossein Sadeghi discovered that reduced levels of CYP3A4 are significant in the development of colorectal cancer.^[39]^ The enzyme CYP17A1 plays a crucial role in steroid synthesis, regulating levels of glucocorticoids important for immune and stress responses, as well as controlling levels of androgens and estrogens critical for reproductive tissue development and stability.^[40]^ In a study by Jiang Z, it was observed that overexpression of CYP2C9 led to a reduction in invasion and migration of human esophageal squamous cell carcinoma (ESCC) cells.^[41]^ Additionally, Liu X indicated that UGT2B7 and CYP3A4 could serve as potential biomarkers for diagnosing nonalcoholic fatty liver disease and hepatocellular carcinoma.^[42]^

Based on the results of KEGG enrichment analysis and GSEA analysis, HFDP has the ability to modulate the body immune response by regulating the retinol metabolism signaling pathway. Vitamin A refers to all compounds that have the biological activity of retinol. Observations of vitamin A-deficient animals have shown that vitamin A intake is inversely proportional to the risk of cancer.^[43]^ Guo X showed abnormal retinoid metabolism during the occurrence of prostate cancer.^[44]^ Guoshu Bi found that regulating iron death can be used as a potential treatment for tumor inhibition. Tumor cells can be protected from lipid peroxidation and cell death caused by different iron death inducers through the action of retinol saturase (RETSAT).^[45]^

The primary active constituents of HFDP for enhancing immunity and possessing multiple targets include cerevisterol, trametenolic acid, and jaranol. Both cerevisterol and trametenolic acid belong to triterpenoids. According to Chien-Liang Chao, the poria triterpenoids have the ability to boost nonspecific immunity by stimulating the secretion of interferon γ (IFN-γ).^[46]^ Additionally, the *Poria cocos* extract has a positive impact on immune regulatory activity. Toshihiro Akihisa found that various triterpenoids extracted from Poria poria showed inhibitory effects on the promotion of skin tumors.^[47]^ Zhang L found that astragaloside, a triterpenoid component in *A membranaceus*, can reduce the inflammatory response and improve immune function in rats with experimental periodontitis.^[48]^

## 5. Conclusion

**Figure 7.**
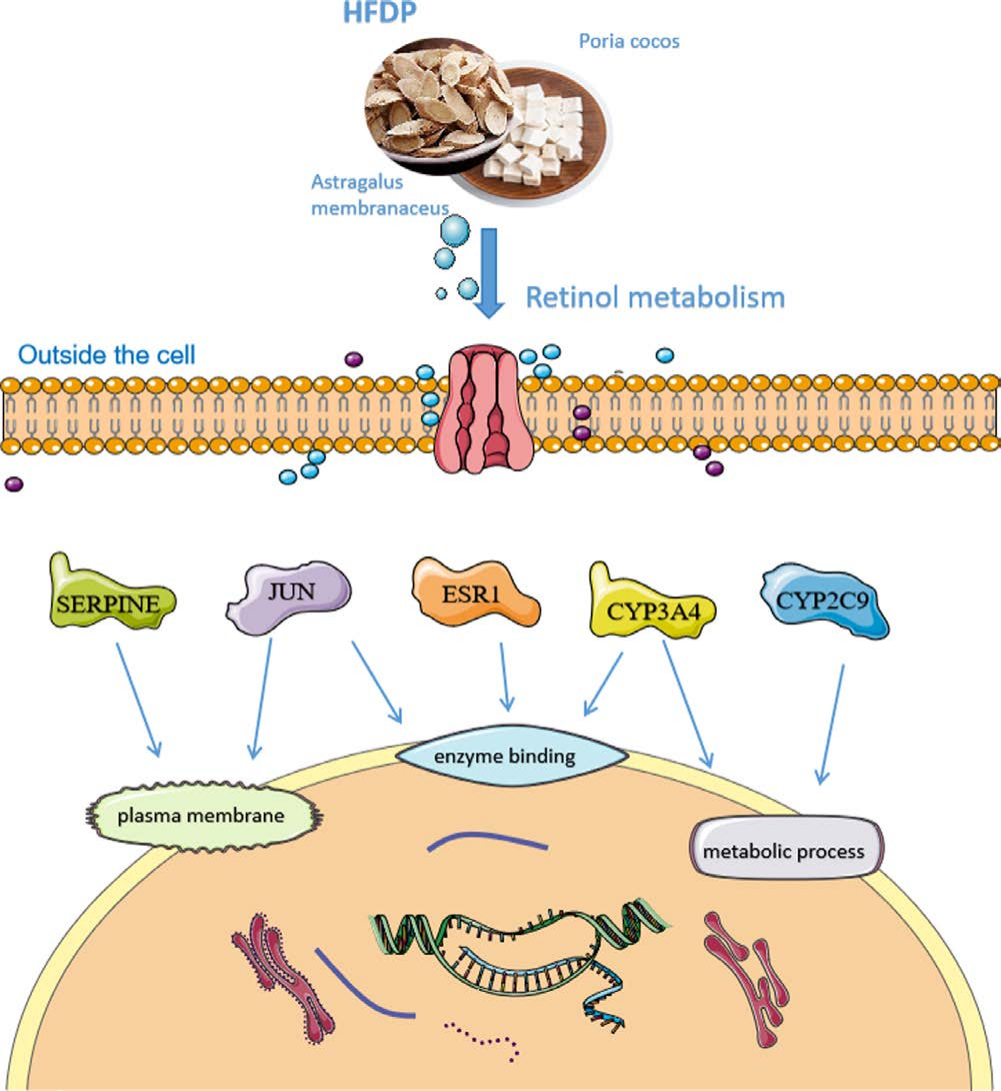
Mechanism of HFDP in the treatment of immunity. HFDP = *Astragalus membranaceus* and Poria cocos drug pair.

In summary, HFDP can mediate metabolism, steroid hormone biosynthesis, and other signal pathways by acting on ESR1, JUN, SERPINE, CYP2C9, CYP3A4, and other triterpenoid components such as cerevisterol and trametenolic acid, thereby regulating immunity. A possible mechanism is shown in Figure 7. However, this study only discussed the active ingredients and mechanisms of HFDP in improving immunity from the perspective of data mining. Subsequently, we will assess the impact of the active components found in HFDP on enhancing immunity through in vitro cell experiments and in vivo animal experiments to confirm the intervention effectiveness. Lentivirus transfection and other methods were used to silence the genes corresponding to the core proteins to verify the signaling pathway for improving immunity. Furthermore, proteomics, complete transcriptomics, metabolomics, and other high-throughput analysis methods were employed to investigate the mechanism of HFDP in enhancing immunity from different perspectives and facets, aiming to offer a more empirical foundation for enhancing immunity through TCM.

## Acknowledgments

We sincerely thank the public databases mentioned in this article for generously sharing a large amount of data.

## Author contributions

**Data curation:** Yuting Bai.

**Funding acquisition:** Yi Nan, Ling Yuan.

**Methodology:** Yuting Bai, Yuhua Du, Xuelan Feng.

**Resources:** Yuting Bai, Yuanyuan Feng.

**Software:** Yuting Bai, Jianjun Zhao, Xilong Jiang.

**Supervision:** Yi Nan, Ling Yuan.

**Validation:** Yuting Bai.

**Visualization:** Yuting Bai, Guoqing Chen, Shicong Huang.

**Writing – original draft:** Yuting Bai, Na Ning.

**Writing – review & editing:** Yi Nan, Ling Yuan.
